# Supplementary material for: Structural insights into SorCS2–Nerve Growth Factor complex formation
Source: Nat Commun. 2018 Jul 30;9:2979. doi: 10.1038/s41467-018-05405-z (PMC6065357; doi:10.1038/s41467-018-05405-z)
Supplement: Supplementary file 1 — Supplementary Information [file 41467_2018_5405_MOESM1_ESM.pdf]

Structural insights into SorCS2 – Nerve Growth Factor complex formation

Nadia Leloup et al.

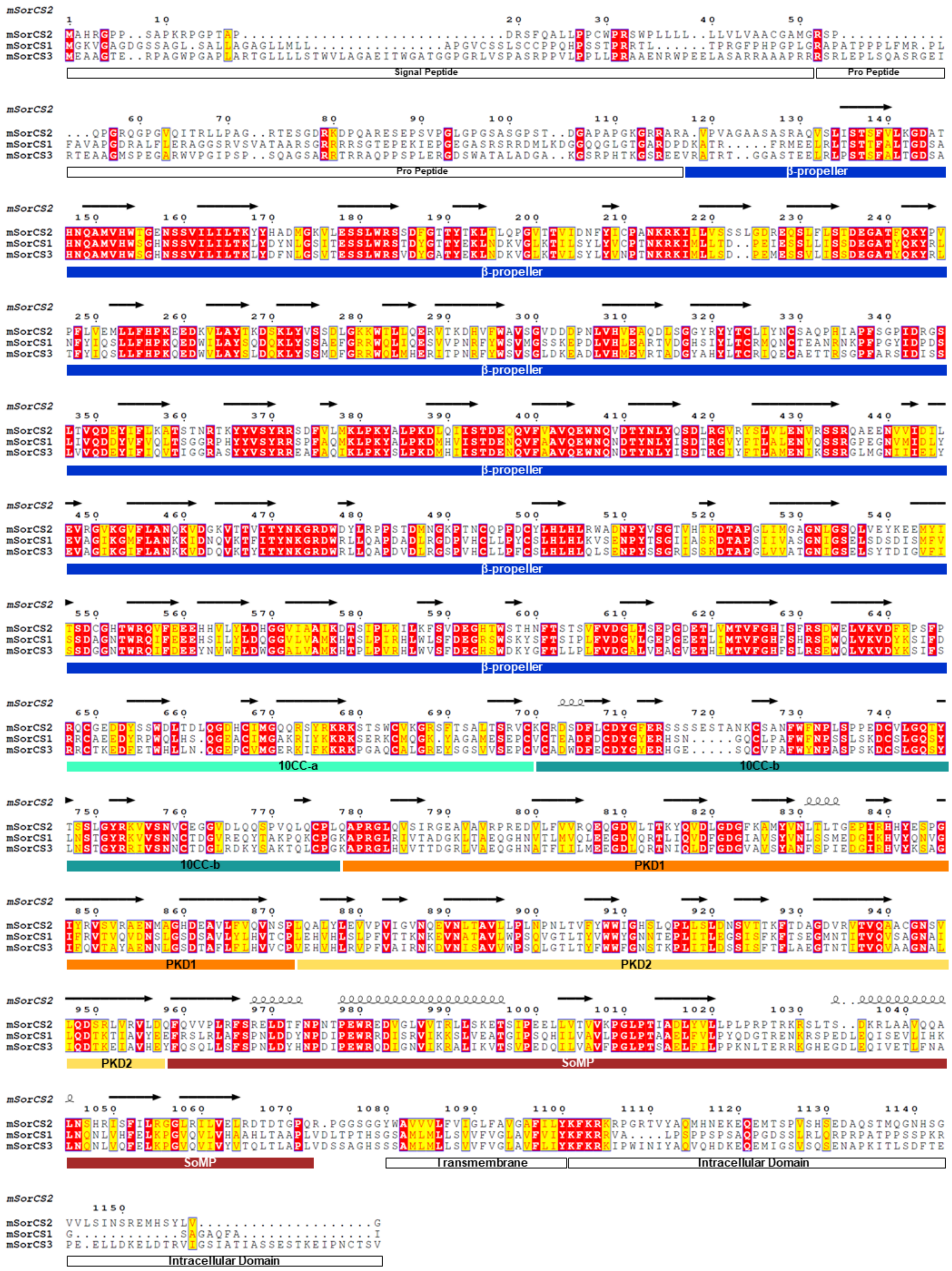

Supplementary Figure 1 | **Ectodomains of SorCS members have a similar structure.** Aligned sequences of all mouse SorCS familiy members (SorCS1-3), identical residues with red background and conserved residues with yellow background. Numbering corresponds to full-length mouse SorCS2. Secondary structure assignments derived from the structure of sSorCS2 are displayed above the alignment. Domains are indicated by colored rectangles below the alignment (colored according to fig 1A). The figure was prepared with ESPript (espript.ibcp.fr/ESPript/cgi-bin/ESPript.cgi).

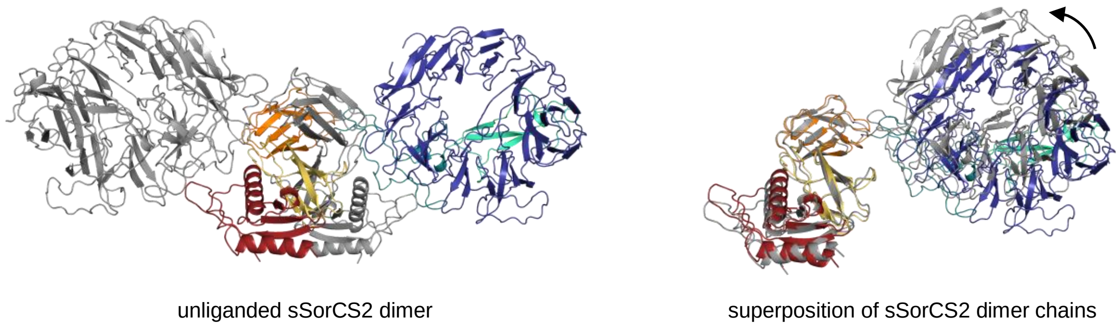

Supplementary Figure 2 | **The two SorCS2 chains of the unliganded dimer adopt slightly different conformations.** Cartoon representation of the two chains of sSorCS2 with chain A colored by domain and chain B in grey. The superposition of the two dimer chains on the basis of the two PKD domains (right) shows that both the angles between PKD1 and the β-propeller, and PKD2 and the SoMP are different in the two chains, indicating some flexibility of the chains within the dimer. The repositioning of the β-propeller, by a 11 Å translation and a 11-degree rotation, is indicated by an arrow.

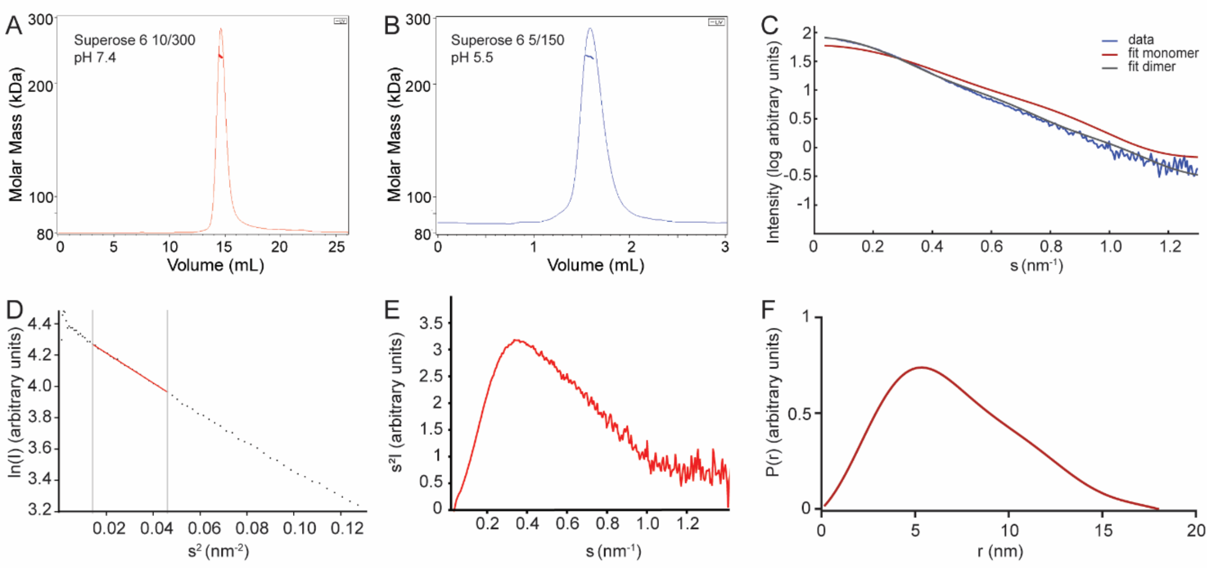

Supplementary Figure 3 | **Solution experiments confirm that sSorCS2 is a dimer.** A-B, Size exclusion chromatography coupled multi angle light scattering of sSorCS2 at pH 7.4 (A) and pH 5.5 (B). A, UV trace and molar mass distribution of sSorCS2 from HEK-ES (red) at pH 7.4 on a Superose 6 10/300 column indicates a molar mass of 236 ± 2 kDa for sSorCS2, corresponding well to the expected mass of 235 kDa (including oligomannose glycosylation) for a sSorCS2 dimer. B, UV trace and molar mass distribution of sSorCS2 from HEK-E (blue) at pH 5.5 on a Superose 6 5/150 column indicates a molar mass of 237 ± 3 kDa for sSorCS2, corresponding reasonably well to the calculated molar mass of 246 kDa (including hybrid glycosylation) of a sSorCS2 dimer. C, Overlay of wt sSorCS2 size-exclusion coupled SAXS trace at pH 5.5 with Crysol fits based on the structures of the sSorCS2 dimer and a single chain. The better fit of the SAXS data to that calculated from the dimer structure (χ^2 = 1.6) compared to that of a single chain (χ^2 = 7.2) indicates that sSorCS2 in solution is predominantly a dimer. The sSorCS2 dimer conformation from the sSorCS2-NGF complex also fits well to the SAXS data (χ^2 = 1.2). D, Guinier plot. The two grey bars indicate the limits of the data points used for determination of the radius-of-gyration, Rg, and the extrapolated intensity at zero scattering angle, I(0). The red line indicates the fit. The Rg determined for sSorCS2 is 5.56 ± 0.02 nm. E, The Kratky plot shows that sSorCS2 is folded. F, The pair distance distribution function P(r) of sSorCS2 is typical of a multidomain protein and indicates a maximum particle size Dmax of 18.0 ± 1.0 nm and a Porod volume of of 419 kÅ^3, which corresponds to a molar mass (Mm) of 246 kDa. This is consistent with the predicted molecular weight of 246 kDa (including hybrid glycosylation) for a sSorCS2 dimer.

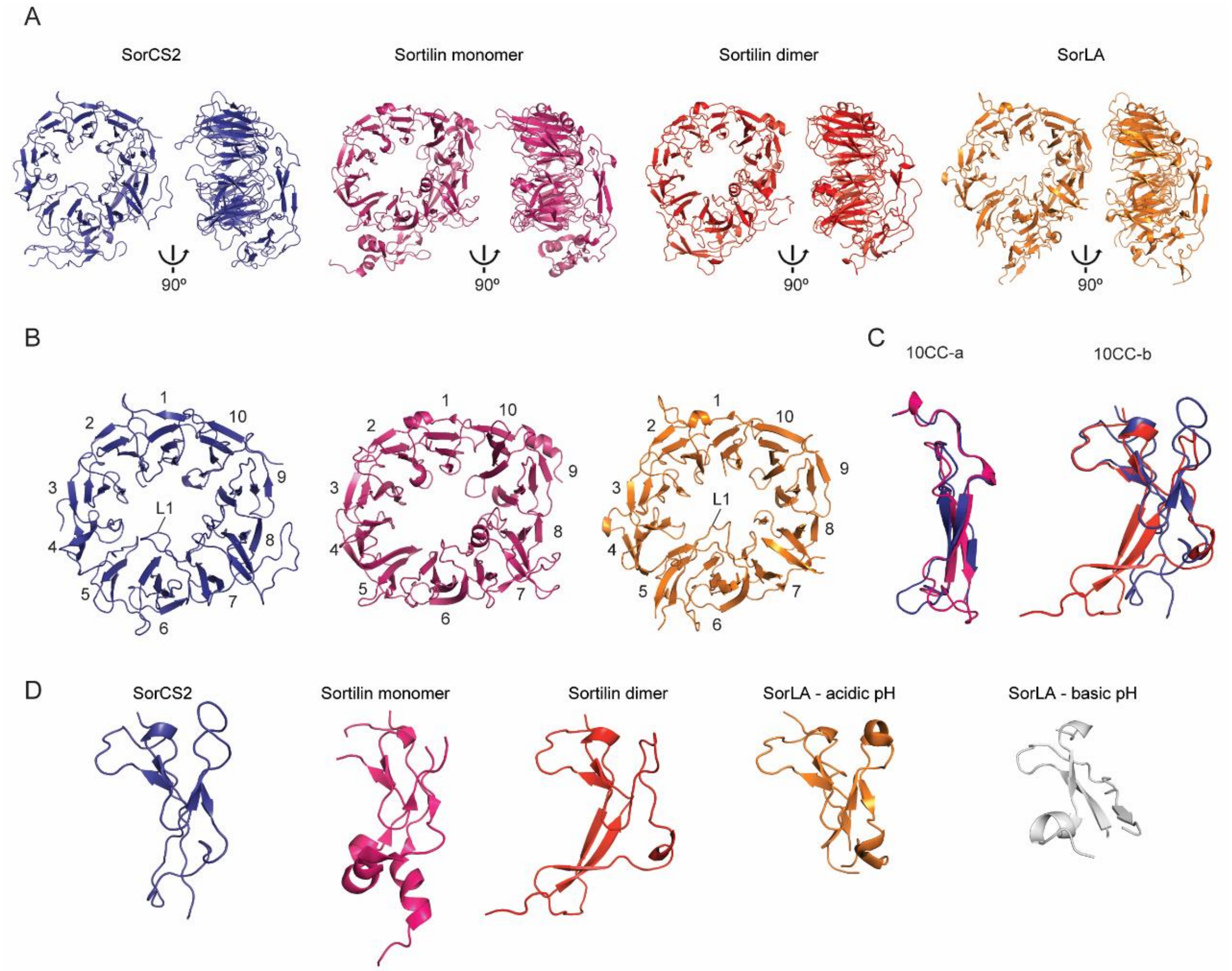

Supplementary Figure 4 | **The SorCS2 VPS10p subunit is similar to those of Sortilin and SorLA.** A, Cartoon representation of the VPS10p subunits of receptors belonging to the VPS10p family: sSorCS2 (blue), sSortilin monomer (pink, pdb-id 3f6k [14]), sSortilin dimer (red, pdb-id 5nmt [17]) and SorLA (orange, pdb-id 3wsx [15]). B, Zoom on the β-propeller of the different receptors (colors same as A). The L1 loop [15] partially shields the β-propeller tunnel of sSorCS2 (blue) and sSorLa, but is much shorter in sSortilin (pink, pdb-id 3f6k [14]). C, The conformation of the 10CC-a domain of sSorCS2 (left panel) is similar to the conformation of the 10CC-a domain of Sortilin monomer (pdb-id 3f6k, rmsd of 1.2 Å over 46 Cα atoms). The 10CC-b domain of sSorCS2 (right panel) adopts a position (see A) and conformation similar to the 10CC-b of the Sortilin dimer (pdb-id 5nmt, rmsd of 1.7 Å over 37 Cα atoms) (colors same as A). D, The 10CC-b domains of the VPS10p family members, including SorLA crystallized at basic pH (grey, pdb-id 3wsz [15]), are shown (colors same as A).

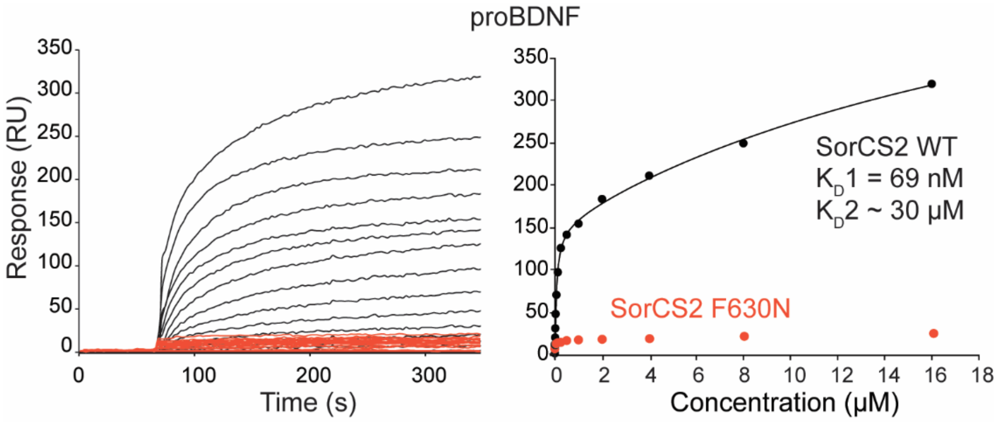

Supplementary Figure 5 | **ProBDNF binds at the same site as NGF in the sSorCS2-NGF structure.** ProBDNF binds to SorCS2 with an affinity of 69 ± 1 nM (n=2), in the same affinity range as proNGF (43 ± 12 nM (n=3), Fig. 4a). The binding is disrupted by introducing an N-linked glycan in the NGF binding site on sSorCS2 (F630N, Fig. 3d). At high concentrations of sSorCS2, a second event is observed, possibly due to sSorCS2 oligomerization.

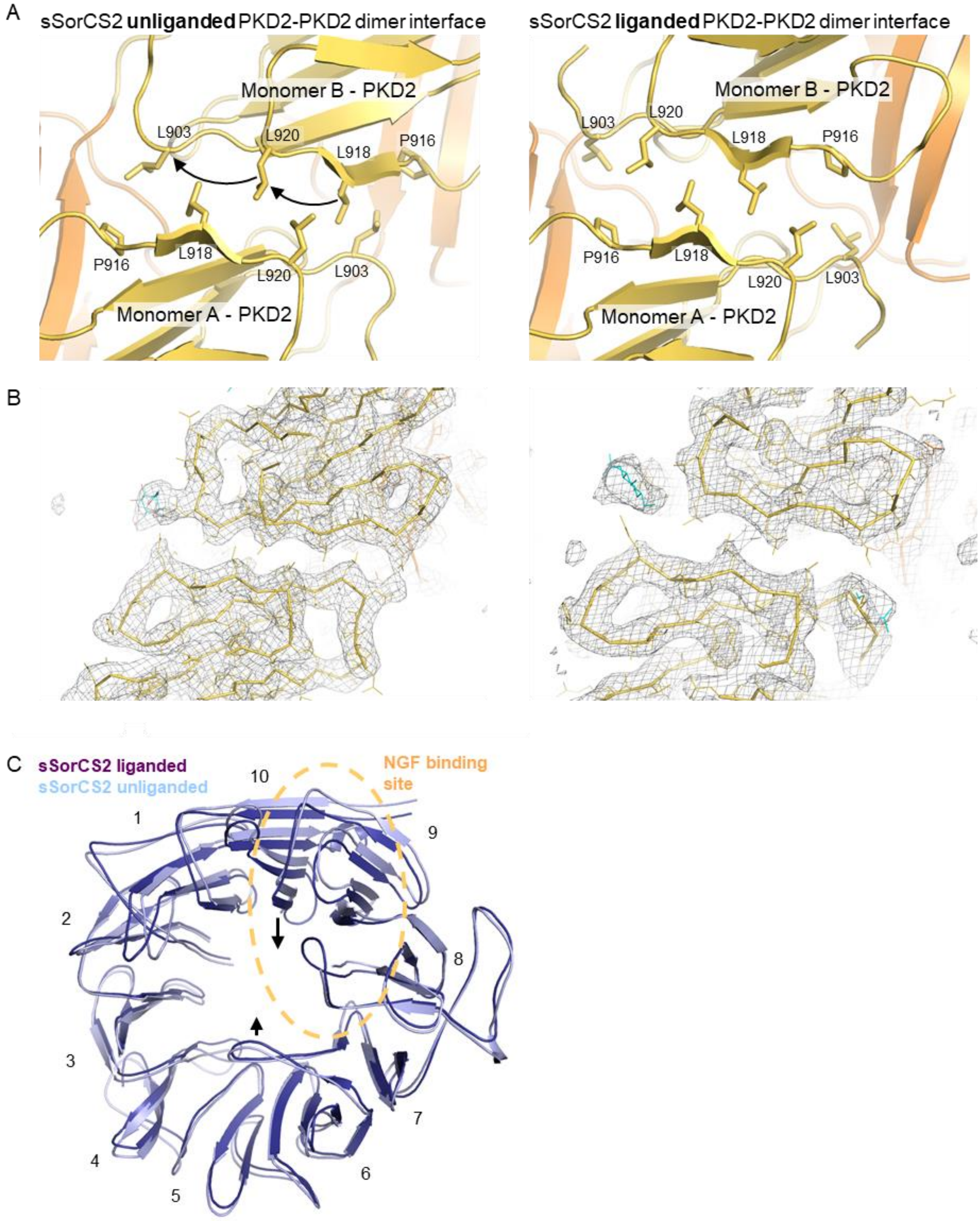

Supplementary Figure 6 | **Conformational plasticity of unliganded and ligand bound sSorCS2.** A, The PKD2-PKD2 sSorCS2 dimer interface has a different conformation in the unliganded and NGF bound structures. The PKD2 domains rotate (by 30.5 degrees) and translate (by 6.6 Å), and as a result slide antiparallel with respect to each other. This movement is illustrated by leucines 903, 918 and 920 that jump one register in the hydrophobic interface. This stepwise conformational change is indicated by two arrows in which leucine positions 903 and 920 in the unliganded sSorCS2 have been taken over by leucines 920 and 918 in the liganded (i.e. the NGF bound) conformation, respectively. B, $2mF_{obs}$-DF_{calc} electron density maps contoured at 1 sigma (grey), with similar views as in A at the PKD2 dimer interface (unliganded sSorCS2, left panel and the liganded sSorCS2, right panel). C, The

sSorCS2 β-propeller undergoes a compaction upon NGF ligand binding in which blades 9 and 10 move towards opposing blade 5 (conformational change is indicated by arrows). Cartoon representation of a superposition of the sSorCS2 β-propeller in the unliganded (light blue) and NGF bound conformation (dark blue). β-propeller blades are numbered. The NGF binding site is indicated by an oval shape.

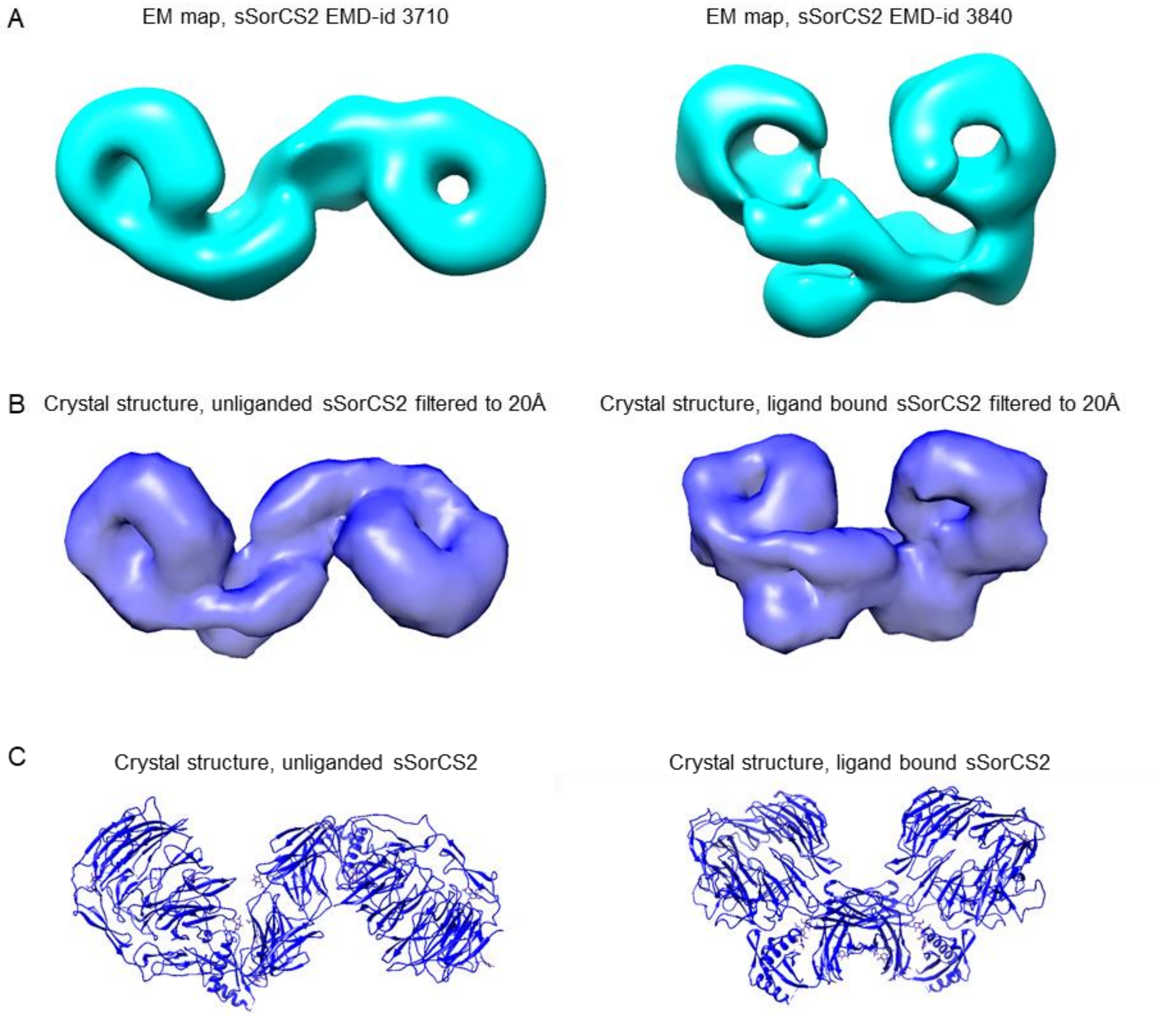

Supplementary Figure 7 | s**SorCS2 has substantial conformational plasticity.** A, Low resolution negative stain electron microscopy (EM) reconstructions of sSorCS2 revealed two conformations (Electron Microscopy Databank (EMD) id 3710 and 3840) [19]. B-C, The crystal structures of unliganded sSorCS2 (left) and NGF-bound sSorCS2 with NGF removed for clarity (right) are shown filtered to 20 Å resolution (B) or as cartoon representation (C). The two sSorCS2 conformations identified by EM fit reasonably well with the two sSorCS2 conformations identified in this study but the differences between the EM maps are larger than the differences between the crystal structures.

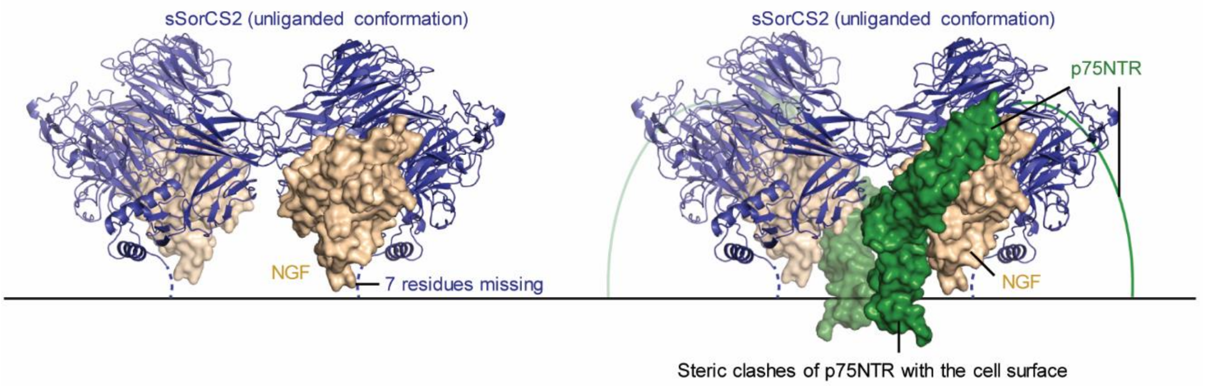

Supplementary Figure 8 | **The SorCS2 conformational rearrangement is required to prevent steric hindrance during SorCS2-(pro)NGF-p75NTR ternary complex formation.** Hypothetical model of NGF in complex with the unliganded conformation of sSorCS2 (left panel). Surface representation of the NGF dimer (beige) bound to the unliganded conformation of SorCS2 in cartoon representation (blue). In this sSorCS2 conformation the β-propeller is oriented with its ligand binding top face close to the cell surface. If (pro)NGF would bind to sSorCS2 in this conformation, steric hindrance could arise from the cell membrane and (pro)NGF. Hypothetical model of the p75NTR-(pro)NGF-SorCS2 ternary complex with sSorCS2 in the unliganded conformation (right panel). Representation as in the left panel with p75NTR in green surface representation. The flexible 61-residue stalk of p75NTR is depicted by the green curved line. In this conformation, the N-terminus of p75NTR would have substantial clashes with the cell membrane, making complex formation impossible.

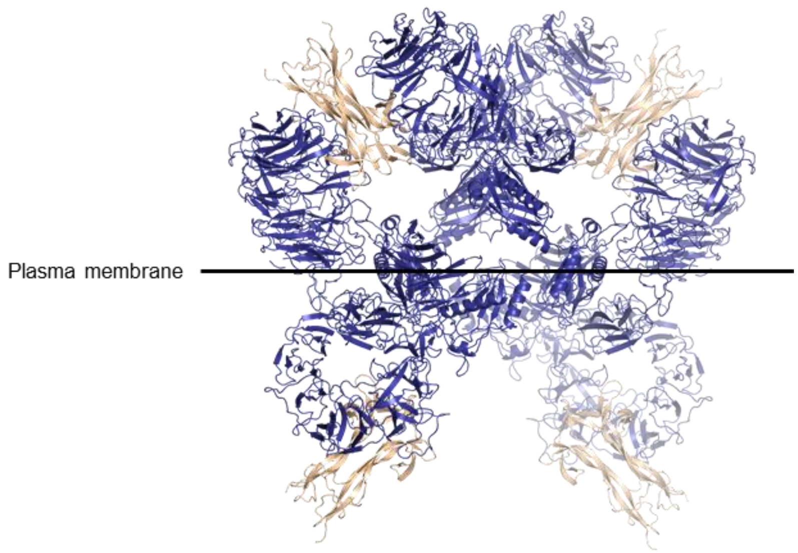

Supplementary Figure 9 | **A second SorCS2 dimer binding to the free NGF site would lead to severe steric hindrance by the plasma membrane.** A straightforward superposition of SorCS2 dimer structure onto the “free” binding site in the crystal shows that a second SorCS2 dimer binding onto the site unoccupied in the crystal would create clashes with the plasma membrane on the cell surface, and a SorCS2-NGF array formation is thus unlikely.

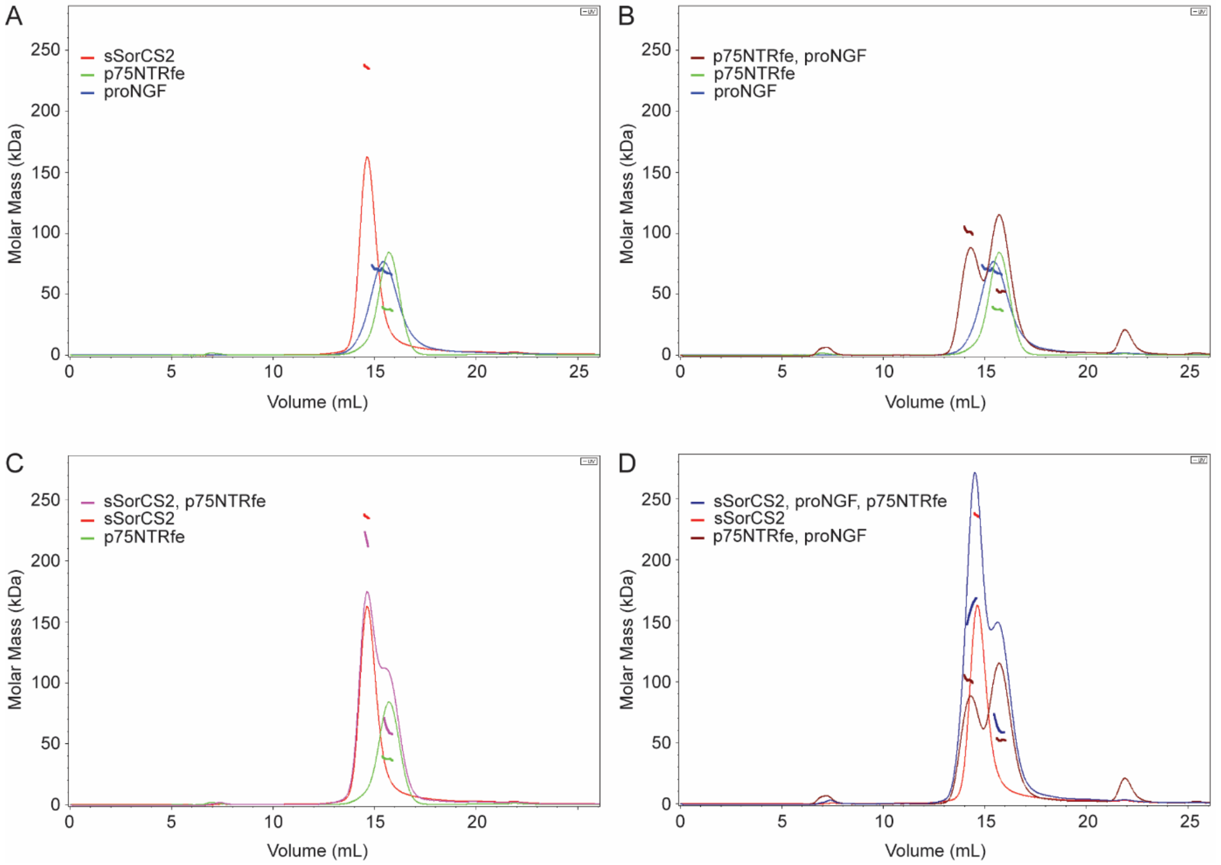

Supplementary Figure 10 | **SEC-MALS analysis of p75NTR, proNGF and SorCS2 does not reveal ternary complex formation.** A-D. Size exclusion chromatography coupled multi angle light scattering of sSorCS2, the full ectodomain of p75NTR (p75NTRfe) and proNGF on their own and mixed together. All experiments were done at pH 7.4 in presence of 2mM CaCl2 on a Superose 6 10/300 column. A, Combined individual UV traces and molar mass distribution of sSorCS2 (red), p75NTRfe (green) and proNGF (blue) show respective measured masses of 236 ± 2 kDa (corresponding to a sSorCS2 dimer see Suppl. Fig. 3), 37 ± 2.0 kDa (which is higher than the calculated 24.7 kDa due to substantial o-glycosylation of the stalk region) and 69 ± 4.7 kDa (slightly higher than the expected 56 kDa for a proNGF dimer). B, Combined individual UV traces and molar mass distribution of p75NTRfe (green) and proNGF (blue), and the p75NTRfe - proNGF mixture (brown) show clear complex formation of p75NTRfe with proNGF. The first peak, shifted to smaller retention volume, has a measured mass of 101 ± 3.0 kDa, which corresponds to a mix of 1:2 (expected 90.7 kDa) and 2:2 (expected 125.4 kDa) p75NTRfe-proNGF complex, as has previously been observed [12]. The second peak has a measured mass of 52 ± 3.4 kDa, which corresponds to an average of the two separate components p75NTRfe and proNGF. C, Combined individual UV traces and molar mass distribution of sSorCS2 (red) and p75NTRfe (green), and the sSorCS2 - p75NTRfe mixture (pink) show no complex formation. The mass of the first and second peaks are averages of different quantities of sSorCS2 and p75NTR, and no peak shifts are apparent. In addition, the elution profile of the sSorCS2 - p75NTRfe mixture is a summation of the elution profiles of the individual components. D, Combined UV traces and molar mass distribution of sSorCS2 (red), the p75NTRfe - proNGF mixture (brown) and the sSorCS2 - proNGF - p75NTRfe mixture (blue) show no ternary complex formation. sSorCS2 is not involved in complex formation while p75NTR and proNGF do interact. The masses of the first and second peaks are averages of different quantities of sSorCS2, p75NTR-proNGF, p75NTR and proNGF and no peak shifts are apparent. In addition, the elution profile of the sSorCS2 - proNGF - p75NTRfe mixture is a summation of the elution profiles of sSorCS2 only and the proNGF - p75NTRfe combination.

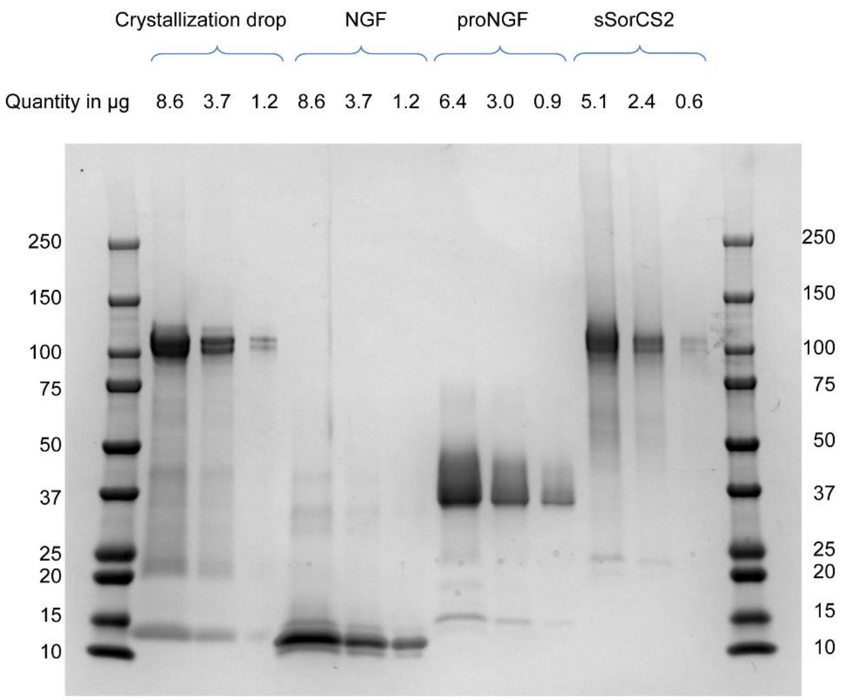

Supplementary Figure 11 | **SDS-Page of the crystallization drop shows processing of proNGF into NGF after three weeks at room temperature.** A band at 13 kDa corresponding to NGF is clearly visible while the proNGF band just below the 37 kDa marker band has disappeared. Quantities for the crystallization drop represent the total quantity of 1:1.1 sSorCS2:proNGF complex set up. The gel was stained by Coomassie Brilliant Blue.

Supplementary Table 1 | **Codon-optimized sequences.**

Codon-optimized cleavage-resistant proNGF DNA sequence

GGATCCGAACCGTACACAGATAGCAATGTCCCAGAAGGAGACTCTGTCCCTGAAGCCCACTGGACTAA
ACTTCAGCATTCCCTTGACACAGCCCTCGCCGCAGCCCGCAGTGCCCCTACTGCACCAATAGCTGCCC
GAGTGACAGGGCAGACCCGCAACATCACTGTAGACCCCAGACTGTTTAAGAAAGCGGCACTCCACTCA
CCCCGTGTGCTGTTCAGCACCCAGCCTCCACCCACCTCTTCAGACACTCTGGATCTAGACTTCCAGGC
CCATGGTACAATCCCTTTCAACAGGACTCACCGGAGCGCGGCCTCATCCACCCACCCAGTCTTCCACA
TGGGGGAGTTCTCAGTGTGTGACAGTGTCAGTGTGTGGGTTGGAGATAAGACCACAGCCACAGACATC
AAGGGCAAGGAGGTGACAGTGCTGGCCGAGGTGAACATTAACAACAGTGTATTCAGACAGTACTTTTT
TGAGACCAAGTGCCGAGCCTCCAATCCTGTTGAGAGTGGGTGCCGGGGCATCGACTCCAAACACTGGA
ACTCATACTGCACCACGACTCACACCTTCGTCAAGGCGTTGACAACAGATGAGAAGCAGGCTGCCTGG
AGGTTCATCCGGATAGACACAGCCTGTGTGTGTGTGCTCAGCAGGAAGGCTACACGACGAGGCGCGGC
CGC

Codon-optimized cleavage-resistant proBDNF DNA sequence

GGATCCGCGCCCATGAAAGAAGTAAACGTCCACGGACAAGGCAACTTGGCCTACCCAGGTGTGCGGAC
CCATGGGACTCTGGAGAGCGTGAATGGGCCCAGGGCAGGTTCGAGAGGTCTGACGACGACATCACTGG
CTGACACTTTTGAGCACGTCATCGAAGAGCTGCTGGATGAGGACCAGAAGGTTCGGCCCAACGAAGAA
AACCATAAGGACGCGGACTTGTACACTTCCCGGGTGATGCTCAGCAGTCAAGTGCCTTTGGAGCCTCC
TCTACTCTTTCTGCTGGAGGAATACAAAAATTACCTGGATGCCGCAAACATGTCTATGAGGGTTCGGG
CCGCCTCCGACCCTGCCCGCCGTGGGGAGCTGAGCGTGTGTGACAGTATTAGCGAGTGGGTCACAGCG
GCAGATAAAAAGACTGCAGTGGACATGTCTGGCGGGACGGTCACAGTCCTAGAGAAAGTCCCGGTATC
CAAAGGCCAACTGAAGCAGTATTTCTACGAGACCAAGTGTAATCCCATGGGTTACACCAAGGAAGGCT
GCAGGGGCATAGACAAAAGGCACTGGAACTCGCAATGCCGAACTACCCAATCGTATGTTCGGGCCCTT
ACTATGGATAGCAAAAAGAGAATTGGCTGGCGATTCATAAGGATAGACACTTCCTGTGTATGTACACT
GACCATTAAAAGGGG

Supplementary Table 2 | **Primers used for DNA amplification.**

Construct	Direction	Sequence
**sSorCS2 WT**	FW	AATAATGGATCCATGGGGCGCTCCCCCCAG
**sSorCS2 WT**	RV	AATAATGCGGCCGCCTGAGGACCTGTGTCTGTGTCC
**sSorCS2 F630N**	FW	CTCTGACTGGGAGCTGGTCAAG
**sSorCS2 F630N**	RV	CGGTTGCTGATGTGACCAAAGAC
**p75NTRfe**	FW	AATAATGGATCCAAGGAGACATGTTCCACAGGCATG
**p75NTRfe**	RV	AATAATGCGGCCGCGTCAGCGGTGCCTCGGG
**proNGF**	FW	AATAATGGATCCGAACCGTACACAGATAGCAATGTC
**proNGF**	RV	AATAATGCGGCCGCGCCTGCTGTCGTAGCCTTCCTGCTGAGC
**proBDNF**	FW	AATAATGGATCCGCGCCCATGAAAGAAGTAAACGTC
**proBDNF**	RV	AATAATGCGGCCGCTCTTCCCCTTTTAATGGTCAGTGTAC
