# Supplementary material for: Structural insights into SorCS2–Nerve Growth Factor complex formation
Source: Nat Commun. 2018 Jul 30;9:2979. doi: 10.1038/s41467-018-05405-z (PMC6065357; doi:10.1038/s41467-018-05405-z)
Supplement: Supplementary file 3 — Description of Additional Supplementary Files [file 41467_2018_5405_MOESM3_ESM.pdf]

Description of Additional Supplementary Files

File Name: Supplementary Movie 1
Description: The full-length extracellular segment of the SorCS2 receptor undergoes a large and global conformational change upon NGF ligand binding.
